# Traumatic injuries detected at slaughter in cattle: impact of production system and season on animal welfare and meat condemnation in Sweden

**DOI:** 10.1186/s13028-025-00804-x

**Published:** 2025-04-11

**Authors:** Josefine Jerlström, Ann-Kristina Lind, Cecilia Lindahl, Charlotte Berg, Anna Wallenbeck

**Affiliations:** 1 Department of Applied Animal Science and Welfare, Faculty of Veterinary Medicine, Swedish University of Agricultural Sciences, Uppsala, Sweden; 2 Department of Research and Development, Växa, Uppsala, Sweden; 3 Department of Agriculture and Food, RISE Research Institutes of Sweden, Uppsala, Sweden

**Keywords:** Beef cattle, Carcass damage, Dairy cattle, Economic impact, Food loss, Lesions, Meat inspection, Organic

## Abstract

The purpose of ante- and post-mortem inspections at slaughterhouses is to ensure that meat and other relevant food products of animal origin are safe for human consumption. However, these inspections can also be useful for detecting animal health and welfare issues. In cattle, traumatic injuries from on-farm incidents, transport or handling at the slaughterhouse are indications of both reduced animal welfare and increased risk of food waste, ultimately resulting in economic losses for both farmers and slaughterhouses. This observational study aimed to investigate the prevalence and seasonal variation of traumatic injuries in cows and heifers reared on organic and conventional farms in Sweden. The study includes slaughter remarks and condemnations from meat inspection data from 336,071 animals slaughtered between 2020 and 2022. Two types of injuries were analysed: “chronic traumatic injuries” (CTI) sustained on-farm and “acute traumatic injuries” (ATI) sustained during transport or at the slaughterhouse. Logistic regression models were developed to assess the influence of production system and season. Results show a higher prevalence of CTI in animals from conventional farms (9.8%) compared to organic farms (6.9%; P < 0.001), which may indicate that animals from organic farms are managed and handled in a way that makes them better prepared for challenges that they are later exposed to on-farm prior to slaughter. ATI were more frequent in animals from organic farms during the grazing period (interaction between production system and season: P = 0.002), which may indicate that animals from organic farms find the transition to the slaughterhouse environment more abrupt and stressful during the grazing period. Condemnations due to injuries were significantly higher for animals with CTI or ATI compared to animals without these specific remarks. These findings highlight the importance of pre-slaughter management, both on-farm and at the slaughterhouse, and slaughterhouse design in improving animal welfare and reducing food as well as economic losses associated with carcase condemnations.

## Findings

Animal welfare, i.e. the subjective experience of the animal, biological function and ability to adapt to the environment in which it is kept [1], includes all aspects of the animal's life. Public interest in animal welfare has increased over the last decades [2, 3], including the welfare of animals at slaughter. In Sweden, around 400,000 cattle are slaughtered annually. Extensive research affirms the relationship between pre-slaughter experiences, stress, animal handling, and meat quality. These factors include earlier human-animal interactions, transportation effects, time spent in lairage and facility design [4–9]. Accordingly, slaughterhouse staff observe differences in how animals cope and behave during handling at slaughter, which can be influenced by factors such as the on-farm production system (e.g. conventional vs organically certified farms), season (e.g. pasture vs indoor housing periods) and herd origin. All food-producing animals in Europe, including Sweden, are subject to official ante- and post-mortem (meat) inspections at slaughter [10]. At these inspections, the official veterinarian, employed by the Swedish Food Agency, may make decisions such as partial or total condemnation if the meat is deemed unfit for human consumption. A recent study found that the primary reason for partial condemnation of cattle carcases in Sweden was traumatic injuries sustained on the farm [11]. These types of injuries, as well as more recent bruises, are important indicators of poor animal welfare but also have financial consequences for both slaughterhouses and farmers, leading to part or whole carcase condemnations [5, 12–14]. Additionally, such condemnations contribute to food waste, as injured or damaged meat is excluded from the supply chain and deemed unsuitable for human consumption. This observational study aimed to investigate the prevalence and seasonal variation of chronic traumatic injuries (CTI) and acute traumatic injuries (ATI) (i.e. physical injuries, including bruises, fractures, cuts, and hematomas), among cows and heifers, reared on conventional and organic farms in Sweden. Older injuries occurring on the farm are classified as CTI, whereas ATI refers to more recent injuries sustained during transport or at the slaughterhouse.

Information on meat inspection data, i.e. slaughter remarks and carcase condemnation on cows and heifers of dairy and beef breeds slaughtered at Swedish slaughterhouses were provided by the cattle farmer’s association Växa, which routinely collects information for the Swedish dairy and beef recording schemes. Two slaughter remarks of specific interest for animal welfare were identified, CTI and ATI, originating from routine veterinary post-mortem examinations of carcases conducted by the Swedish Food Agency. However, there are no precise guidelines in the inspection instructions for when an acute injury is considered to develop into a chronic one [15]. Only cows and heifers were included, as it is mandatory to keep animals of these categories on pasture during the vegetative season according to Swedish animal welfare legislation [16, 17], enabling assessment of differences between pasture and indoor seasons. The final data set included information on 336,071 slaughtered cows and heifers of both beef and dairy breeds slaughtered 2020–2022 (116,512, 106,390 and 113,169 in the year 2020, 2021 and 2022 respectively), representing 56.2% of all cows and heifers slaughtered in Sweden during the period [18]. Of these, 12.7% originated from farms that were organically certified according to KRAV’s standards [19] (the main organic label in Sweden). Data editing, calculation of descriptive statistics and statistical analyses were performed using Statistical Analysis Software (SAS) version 9.4 (SAS Institute, Inc., Cary, NC). Differences in the prevalence of CTI and ATI between production systems and by season were analysed with logistic regression using PROC GLIMMIX, binomial distribution and logit link. The model included the fixed effects of the production system (organic, conventional), slaughter year (2020, 2021, 2022), slaughter month (12 classes, January – December), animal category (cow, heifer) and the interaction between the production system and slaughter month. Moreover, the model included slaughter weight as a continuous covariate (adjusting for the size of the animal including partial breed effects between heavier and lighter breeds) and the fixed random effect of slaughterhouse nested within slaughter year (including both effects such as management and size of the enterprise as well as geographic location).

In total, 9.4% of carcases from cows and heifers had the remark CTI (6.9% in animals from organic farms and 9.8% of animals from conventional farms) and 1.0% ATI (1.2% in animals from organic farms and 1.0% of animals from conventional farms). Carcase weight, conformation and fatness scores were numerically higher in animals originating from conventional compared to animals from organic farms (Table 1). The amount of condemned meat was higher in carcases with the remark CTI compared to carcases without this specific remark (mean 23.2 kg and 5.1 kg respectively: P < 0.05 with t-test). Similar results were found for the remark ATI with an average of 15.1 kg vs. 6.7 kg respectively (P < 0.05 with t-test). This secondary finding of the present study confirms previous research, emphasising that injuries and bruises sustained during pre-slaughter and slaughter handling are not only indicators of poor animal welfare but also have economic implications for both farmers and slaughterhouses due to carcase condemnations [5, 20–22].

**Table 1:** Carcase weight, conformation, and fat score in cows and heifers from certified organic and conventional farms

	Organically certified farms		Conventional farms	
	n = 42,517		n = 293,554	
	Mean	SD	Mean	SD
Carcase weight (kg)	305.6	52.62	318.1	56.55
Carcase conformation score^1^	4.3	4.89	5.0	5.48
Carcase fatness score^2^	7.0	2.14	7.6	2.28

*SD* standard deviation

^1^Conformation score 3 = P + , 4 = O-, 5 = O, 6 = O + , 7 = R- according to the EUROP carcase classification scheme, with higher scores for more developed muscles

^2^Fatness score 7 = ”3- “, 8 = ”3″, 9 = ”3 + ”, 10 = ”4- “, 11 = ”4″ according to the EUROP carcase classification scheme, with higher scores for more fat on the carcase

**Fig. 1:**
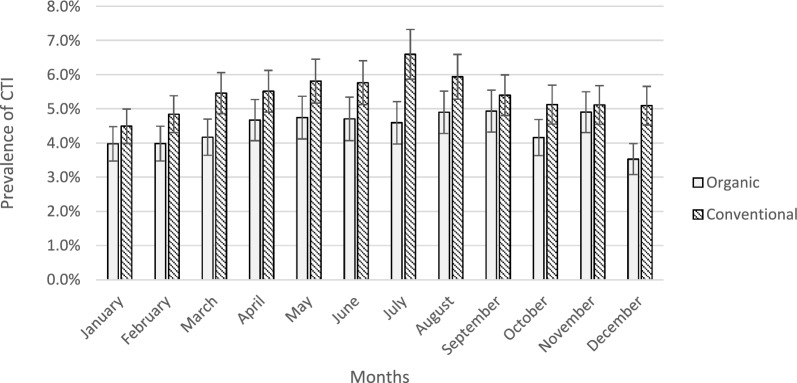
Prevalence of chronic traumatic injuries (CTI) in carcases from organic and conventional farms (2020–2022). The graph shows the least square means (± standard error) of the slaughter remark CTI that occurred on-farm in carcases from cows and heifers slaughtered 2020–2022 originating from organic (n = 42,517) and conventional (n = 293,554) farms

A lower proportion of cows and heifers from organic herds was identified with the remark CTI compared to those from conventional farms. This trend remained consistent across all seasons throughout the year (F = 95.9, P < 0.001; Fig. 1). Differences in management practices between conventional and organic dairy and beef farms in Sweden primarily relate to preventive animal health management (including parasite control), feed composition (notably the restriction on protein feed availability) and animal handling practices at the time of slaughter (Table 2) [19]. While access to pasture is a major distinction between conventional and organic farming in many countries, this is not the case in Sweden, where pasture access is mandatory for both beef and dairy farms [16, 17]. However, KRAV regulations impose stricter requirements regarding, for example, the minimum hours per day animals must spend on pasture and the minimum duration of the pasture season [19]. Regardless of production system, pasture season varies with climate and isthus the vegetative season across Sweden, with shorter seasons in the north and longer seasons in the south. This was partly adjusted for in the statistical analyses by including slaughterhouses nested within the slaughter year as a random effect. The difference in the prevalence of CTI between cows and heifers from organic and conventional farms (6.9% vs. 9.8%) may indicate differences in management and handling practices. Animals from organic farms might be better prepared to handle the on-farm challenges they face prior to slaughter.

**Table 2:** Overview of selected slaughter regulations for KRAV certified, EU organic, and conventional farms (2022)

	KRAV-certified	EU-organic	Conventional
Free access to roughage	Yes	No	No^a^
Maximum transport 8 h	Yes	Yes	Yes
Use of electric prods allowed	No	Yes^b^	Yes
Stay overnight at the slaughterhouse	Yes^c^	Yes	Yes
Stunning prior to exsanguination	Yes	Yes	Yes

^a^Required only during the night

^b^Not allowed at loading or unloading

^c^Not more than 30% of the animals

**Fig. 2:**
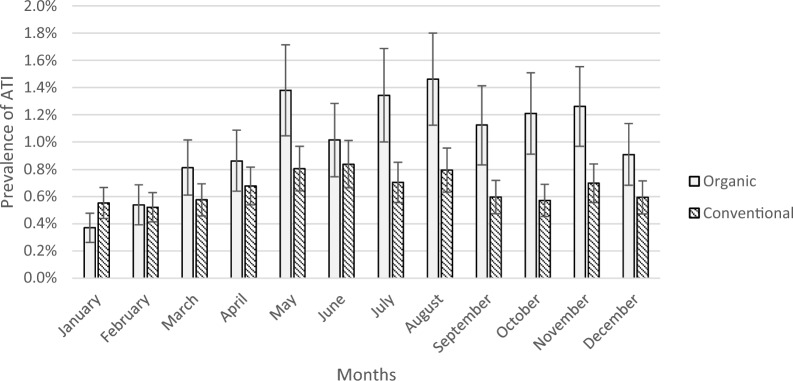
Prevalence of acute traumatic injuries (ATI) in carcases from organic and conventional farms (2020–2022). The graph shows the least square means (± standard error) of the slaughter remark ATI that occurred either during transport or at the slaughterhouse in carcases from cows and heifers slaughtered 2020–2022 originating from organic (n = 42,517) and conventional (n = 293,554) farms

The proportion of cows and heifers with ATI was significantly higher during the grazing period (i.e. when animals are kept on pasture, May – October, depending on geographic location) for animals from organic farms, but not among animals from conventional farms (interaction between production system and season: F = 2.7, P = 0.002; Fig. 2). Animals from conventional farms, which are typically kept outdoors for fewer hours during the day (a minimum of six hours per day are mandatory), might find the transition to the slaughterhouse environment less abrupt and stressful as they spend more time indoors even during the pasture period, potentially explaining the differences in acute traumatic injuries. However, this observational study cannot establish any causal effects (e.g. animals staying overnight, the use of electric prods, etc.), thus further research is needed.

The findings of this observational study provide a starting point for discussions on optimising slaughterhouse facility design in order to prevent on-site injuries and improving pre-slaughter management practices at the farm level to reduce handling-related injuries, thereby enhancing overall animal welfare.

## Data Availability

The datasets used and/or analysed during the current study are available from the corresponding author on reasonable request.

## References

[1] Fraser D, Weary DM, Pajor EA, Milligan BN. A scientific conception of animal welfare that reflects ethical concerns. Anim Welf. 1997;6(3):187–205. 10.1017/S0962728600019795.

[2] European Commission. Attitudes of Europeans towards animal welfare; Special Eurobarometer, 442 Wave EB 84.4. 2016.

[3] European Commission. Attitudes of Europeans towards animal welfare; Special Eurobarometer, 442 Wave EB 99.1. 2023.

[4] Sullivan PA, Davis MK, Nair MN, Hess AM, Mooney DF, Edwards-Callaway LN. Preslaughter factors affecting mobility, blood parameters, bruising, and muscle pH of finished beef cattle in the United States. Transl Anim Sci. 2024;8:txae035. 10.1093/tas/txae035. 38562213 10.1093/tas/txae035 PMC10983080

[5] Wigham EE, Butterworth A, Wotton S. Assessing cattle welfare at slaughter – why is it important and what challenges are faced? Meat Sci. 2018;145:171–7. 10.1016/j.meatsci.2018.06.010. 29982070 10.1016/j.meatsci.2018.06.010

[6] Hultgren J, Wiberg S, Berg C, Cvek K, Lunner KC. Cattle behaviours and stockperson actions related to impaired animal welfare at Swedish slaughter plants. Appl Anim Behav Sci. 2014;152:23–37. 10.1016/j.applanim.2013.12.005.

[7] Grandin T. Factors that impede animal movement at slaughter plants. J Am Vet Med Assoc. 1996;209:757–9. 8756875

[8] Wiberg S. Slaughter – Not only about animals: an interdisciplinary study of handling of cattle at slaughter. Skara: Swedish University of Agricultural Sciences; 2012. Department of Animal Environment and Health, Section of Animal Hygiene.

[9] Gallo CB, Huertas SM. Main animal welfare problems in ruminant livestock during preslaughter operations: a South American view. Animal. 2015;10:357–64. 10.1017/S1751731115001597. 26251114 10.1017/S1751731115001597

[10] European Commission. Commission Implementing Regulation (EC) 2019/627 of 15 March 2019 laying down uniform practical arrangements for the performance of official controls on products of animal origin intended for human consumption in accordance with Regulation (EU) 2017/625 of the European Parliament and of the council and amending Commission Regulation (EC) No 2074/2005 as regards official controls. OJEU. 2019;131:51–95.

[11] Johansson S. Förluster av nötkött på svenska slakterier. [Losses of beef in Swedish abattoirs]. Uppsala: Swedish University of Agricultural Sciences; 2024.

[12] Strappini AC, Metz JHM, Gallo CB, Kemp B. Origin and assessment of bruises in beef cattle at slaughter. Animal. 2009;3:728–36. 10.1017/S1751731109004091. 22444452 10.1017/S1751731109004091

[13] Comin A, Jonasson A, Rockström U, Kautto AH, Keeling L, Nyman A-K, et al. Can we use meat inspection data for animal health and welfare surveillance? Front Vet Sci. 2023;10:1129891. 10.3389/fvets.2023.1129891. 37234071 10.3389/fvets.2023.1129891 PMC10205995

[14] Valkova L, Vecerek V, Voslarova E, Kaluza M, Takacova D. The welfare of cattle, sheep, goats and pigs from the perspective of traumatic injuries detected at slaughterhouse postmortem inspection. Animals. 2021;11:1406. 10.3390/ani11051406. 34069150 10.3390/ani11051406 PMC8156928

[15] Swedish Food Agency. Beslut om kött från tama hov- och klövdjur. https://kontrollwiki.livsmedelsverket.se/artikel/636/beslut-om-kott-fran-tama-hov-och-klovdjur (2012). Accessed 20 Nov 2024.

[16] SFS 1988:534 Djurskyddslag [Swedish Animal Welfare Act]. https://www.riksdagen.se/sv/dokument-lagar/dokument/svensk-forfattningssamling/djurskyddslag-1988534_sfs-1988-534. Accessed 20 Nov 2024.

[17] SFS 1988:539 Djurskyddsförordningen [Swedish Animal Welfare Ordinance]. https://www.riksdagen.se/sv/dokument-lagar/dokument/svensk-forfattningssamling/djurskyddsforordning-1988539_sfs-1988-539. Accessed 20 Nov 2024

[18] Jordbruksverket. Slakt av större lantbruksdjur vid slakteri efter Djurslag, Tabelluppgift och År. [Slaughter of larger farm animals at slaughterhouses by animal species, table data, and ear]. https://statistik.jordbruksverket.se/PXWeb/pxweb/sv/Jordbruksverkets%20statistikdatabas/Jordbruksverkets%20statistikdatabas__Animalieproduktion__Slakt/JO0604A3.px/ (2024). Accessed 11 Nov 2024

[19] KRAV. [Rules and regulations for organic production]. https://www.krav.se/regler/. Accessed 29 Nov 2024.

[20] Alleweldt F, Kara S, Schubert K, Fries R, Großpietsch R. Study on the stunning/killing practices in slaughterhouses and their economic, social and environmental consequences. Final Report, Part 1: Red Meat. European Commission. Brussels: Directorate General for Health and Consumer Protection; 2007.

[21] Jerlström J, Berg C, Karlsson A, Wallenbeck A, Hansson H. A formal model for assessing the economic impact of animal welfare improvements at bovine and porcine slaughter. Anim Welfare. 2022;31:361–71. 10.7120/09627286.31.4.004.

[22] Huertas SM, van Eerdenburg F, Gil A, Piaggio J. Prevalence of carcass bruises as an indicator of welfare in beef cattle and the relation to the economic impact. Vet Med Sci. 2015;1:9–15. 10.1002/vms3.2. 29067169 10.1002/vms3.2 PMC5645810

